# Methacrylate Coatings for Titanium Surfaces to Optimize Biocompatibility †

**DOI:** 10.3390/mi11010087

**Published:** 2020-01-13

**Authors:** Argus Sun, Nureddin Ashammakhi, Mehmet R. Dokmeci

**Affiliations:** 1 Center for Minimally Invasive Therapeutics (C-MIT), University of California, Los Angeles, CA 90095, USA; n.ashammakhi@googlemail.com (N.A.); dokmeci1@gmail.com (M.R.D.); 2 Eidolon Hydros, Buena Park, CA 90622, USA; 3 Department of Bioengineering, University of California, Los Angeles, CA 90095, USA; 4 California Nanosystems Institute, Los Angeles, CA 90095, USA; 5 Department of Radiological Sciences, David Geffen School of Medicine, University of California, Los Angeles, CA 90095, USA

**Keywords:** titanium coating, implanted medical devices, biomaterials, surface chemistry, chemical descriptors, machine learning

## Abstract

Currently, there are more than 1.5 million knee and hip replacement procedures carried out in the United States. Implants have a 10–15-year lifespan with up to 30% of revision surgeries showing complications with osteomyelitis. Titanium and titanium alloys are the favored implant materials because they are lightweight and have high mechanical strength. However, this increased strength can be associated with decreased bone density around the implant, leading to implant loosening and failure. To avoid this, current strategies include plasma-spraying titanium surfaces and foaming titanium. Both techniques give the titanium a rough and irregular finish that improves biocompatibility. Recently, researchers have also sought to surface-conjugate proteins to titanium to induce osteointegration. Cell adhesion-promoting proteins can be conjugated to methacrylate groups and crosslinked using a variety of methods. Methacrylated proteins can be conjugated to titanium surfaces through atom transfer radical polymerization (ATRP). However, surface conjugation of proteins increases biocompatibility non-specifically to bone cells, adding to the risk of biofouling which may result in osteomyelitis that causes implant failure. In this work, we analyze the factors contributing to biofouling when coating titanium to improve biocompatibility, and design an experimental scheme to evaluate optimal coating parameters.

## 1. Introduction

There is an increasing aging population [1,2,3] and an increasing use of implants such as total joint replacement [2,3,4]. The life span of the hip implant [2,5] is around 10–15-years [3,4]. A major problem with such implants is their failure and the consequent need of revision or re-operation which carries major risks to the patients [4]. Implant failure can result from a variety of other reasons that include infection [1,4], failure to integrate [1,6,7], biomechanical problems leading to stress protection and bone resorption [8], and inflammatory reaction to implant degradation products [1,2,3,4,6,7].

Implant infection accounts for up to 30% of revision surgeries show complications with infections [2,3,4]. One of the prophylactic measures to avoid infection is the use of antibiotics loaded into poly(methyl methacrylate) (PMMA), the bone cement applied around the implant [6]. PMMA creates a roughened surface that promotes cell attachment and bone ingrowth, however, even this clinical standard has been found to be problematic and unable to promote sufficient bone ingrowth to prevent long-term implant failure, prompting a need for coating and other surface modifications. The development of total joint replacement (TJR) implants that materially and structurally integrate into the surrounding bone better will help extend implant life. 

TJR implants in development rely on the use of porous foamed titanium surfaces for healing bone to grow into the implant [9]. The development of bioactive coatings for implants can improve the biocompatibility and long-term performance of implants. Cell-adhesive proteins can be crosslinked together through the conjugation of methacrylate monomer groups to lysine side chains. Methacrylate and acrylate monomers are esters with a reactive pi bond between the α and β carbons. Fujisawa and Kadoma [10] showed that varying α substitution (methacrylates are methyl α substituted while acrylates are not) and varying β substitution can have effects on the monomer resistance to hydrolysis. Although methacrylates and acrylates can be polymerized using ultraviolet (UV) light and a photoinitiator, previous work by Khademhosseini et al. [11] has shown that crosslinking methods alternative to UV curing are readily available. In the current work we examine two protein-monomer conjugates that have potential as bioactive coatings, gelatin methacryloyl (GelMA) and methacrylated troponin. Methacrylate linkages can be conjugated to metal surfaces using atom transfer radical polymerization (ATRP). Choi et al. [12] used ATRP to conjugate osteogenic growth factor, bone morphogenetic protein 2 (BMP-2), to a titanium surface with polyethylene glycol (PEG)-methacrylate. Although researchers are hesitant to use ATRP for biomedical applications due to slow reaction times and residual impurities from use of metal catalysts [11,13], Rainier et al. [14,15,16] introduced photo accelerated ATRP which has been joined by metal-free ATRP reactions [13,16]. The attachment of proteins containing integrin-binding RGD sequences facilitate improved biocompatibility through stronger cell attachment to implant surfaces. However, a drawback to creation of a biocompatible surface is that biofouling can occur due to increased nonspecific bacterial attachment. Yarovsky et al. [17] used machine learning to analyze chemical descriptors of polymeric surfaces [18] that have anti-fouling properties. Shiba et al. [19] looked at the effectiveness of coating titanium with peptides with known antimicrobial properties. Studies on protein adsorption to titanium surfaces [20,21] have looked at fibrinogen while lysozyme has been looked at on other surfaces [17].

Using molecular dynamics, properties such as adsorption to metal surfaces can be predicted within reasonable error [22,23,24,25,26]. While molecular descriptors can be reliably calculated using commercial packages [17], there have also been attempts to make structure-based predict bioactivity [27,28,29]. To our knowledge, this is the first attempt to make structure-based predictions of biocompatibility of GelMA and other methacrylatyed proteins on titanium surfaces. In this paper, we use the structures of troponin and collagen to create a generalizable model and workflow to predict biofouling on protein-coated surfaces.

## 2. Biomaterial Modeling

Molecular modeling was performed in chimera and a molecular operating environment (MOE) (Chemical Computing Group, Montreal, Canada). {1, 1, 1} Titanium Lattice was constructed to act as a binding surface, with a cross platform construction of the structural model was important to conserve compute resources. For globular proteins, structures were imported from RCSB and denatured strand proteins were constructed from sequences obtained from the NCBI protein database (see Supplementary Materials).

**Figure 1:**
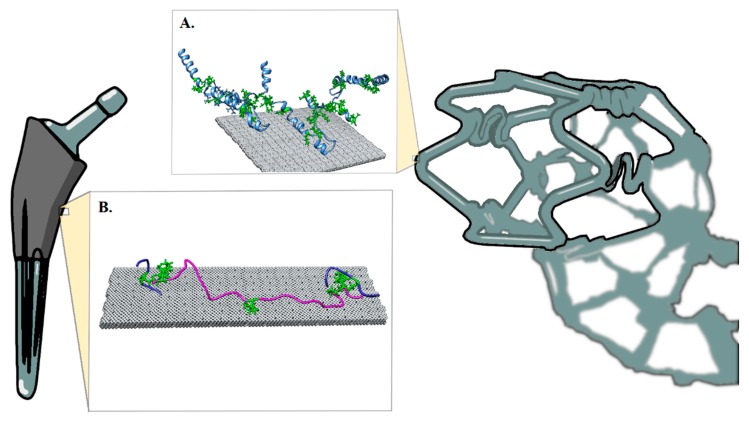
Methacrylated Proteins with lysine residues in green. (**A**) Methacrylated protein for cardiovascular applications (a crosslinked troponin trimer, blue, is shown attached to a gray titanium surface.). (**B**) Gelatin methacrylate (GelMA) trimer (magenta, blue).

**Figure 2:**
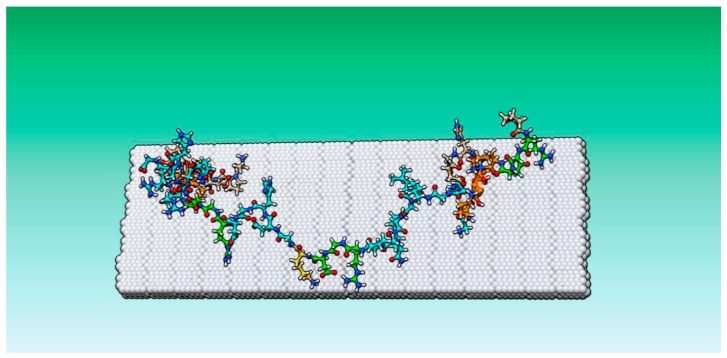
GelMA against titanium surface, this trimer is composed of two shorter gelatin fragments shown in orange (residues 81–89 and 183–192) the longer fragment (173–200) is colored cyan. Lysine residues are colored beige and integrin-binding RGD sequences are green.

In the GelMA model, 60% of lysine residues were modified by methacrylation using the builder function (Figure 1B and Figure 2). The selection of modified residues was randomly assigned, similar to synthetic conjugation of the residues. Multimer assemblies were covalently linked at methacrylated lysines and the resulting structure was energy minimized. 

Protein descriptors were selected from a set of common molecular descriptors found in MOE as well as potentially useful descriptors such as the zeta potential and mobility for coating electrically sensitive surfaces of devices such as electrodes. The structures were converted to .mol2 format in chimera and imported into AlvaDesc (Kode Chemoinformatics, Pisa, Italy). From the pool of 5000+ descriptors calculated for each structure, we selected a set of 421 (see supplemental files). Descriptors were calculated using a multicore Thinkcentre workstation. We allocated runtimes of a few minutes to several hours, and we set a cutoff of 18 hours after which calculation was terminated.

After initial molecular and chemical descriptor (32 descriptors) calculation, we performed an initial principal component analysis (PCA) screen to gauge the data behavior upon dimension reduction. The results were stored in a database file and the file was analyzed using PCA function in MOE.

Subsequently, we calculated the selected pool of 421 descriptors, then exported the results and pooled them into comma-separated files (CSV) format, then inserted the bioactivity data manually. We used Scikit Learn, Numpy, Statsmodel and Pandas packages to complete machine learning on the data. We split the data into 0.25 test set and remainder used the remainder as a training set for PCA. The training and test set was rotated so that the model could be trained and tested on each peptide. We generated a matching matrix and evaluated accuracy using sk-learn and NumPy (see supplemental files). The target data was also treated as continuous and multiple linear regression was used to train a model and make predictions about the coating proteins.

## 3. Results and Discussion

**Figure 3:**
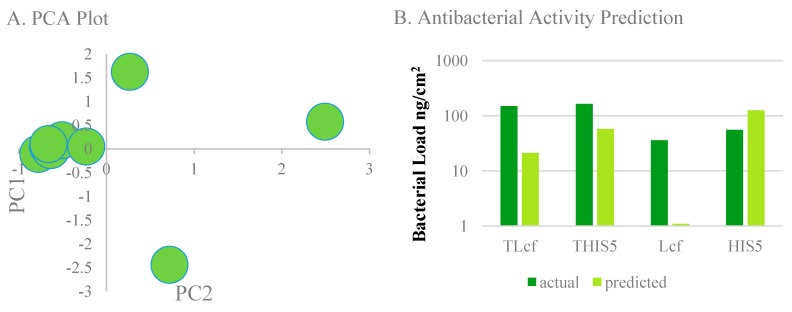
Results on initial datasets, (**A**). principal component analysis (PCA) of 32 descriptor set, (**B**). activity prediction of antibacterial peptides from Shiba et al [19] using multiple linear regression.

We generated a total of 32 molecular and chemical descriptors for each protein or polypeptide conjugate. Descriptors that were consistently low or null valued were excluded, leaving 28 descriptors. The protein set was plotted along the first two principal components seen in Figure 3A. Although the data was not partitioned, a natural division arose along the PC1 axis. 

A selection of the calculated descriptors is shown in Table 1 and Table 2. We selected chemical descriptors that can be compared to experimental values without great difficulty as well as values that are useful for chemical simulation. 

**Figure 4:**
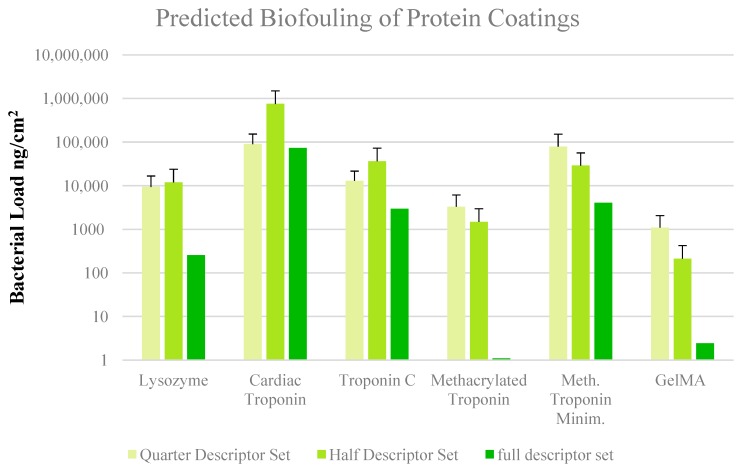
Prediction of antibacterial activity using multiple regression on 421 descriptor set.

**Figure 5:**
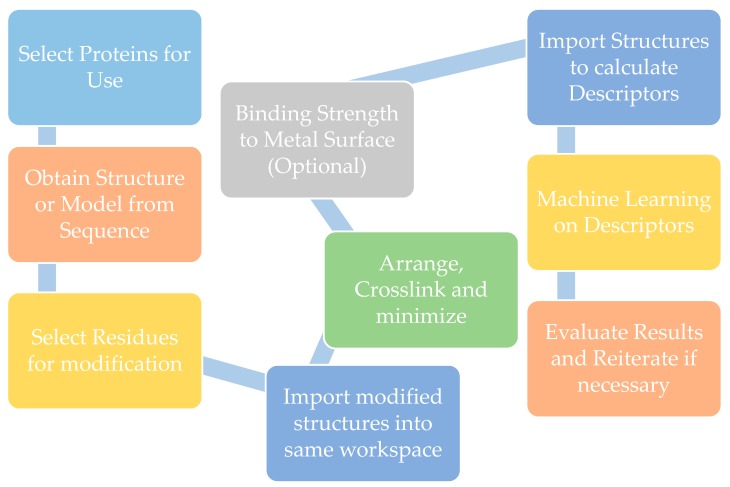
Workflow for evaluating protein coatings for titanium surface.

We then expanded the descriptor set and used the bacterial load in ng/cm^2^ from Shiba et al. [19] as target variables for optimization by PCA. The bacterial load was categorized as high or low based on a threshold of 100 ng/cm^2^. We also left the target data as continuous and performed multiple linear regression (Figure 3B and Figure 4). Through training the algorithm on antimicrobial peptides, we hoped to predict similar structure-based activity in the methcrylated polypeptide polymers. Although the training set produced consistent predictions with high dimensional data, the results on the test set were not robust enough to make accurate predictions when applied to GelMA and other proteins. One reason is the larger size of the proteins when compared to the peptides. During energy minimization, smaller GelMA fragments aggregated in a fashion similar to that seen with hydrophobic sequences of denatured proteins, resulting in precipitation from solution. The difference in energy-minimized structures is seen for methacrylated troponin in Figure 4. The smaller GelMA aggregates may have also distorted the value of some descriptors. Coarse-graining the model can allow model construction without short fragments, though there is a cost in fine-grain structural resolution. Coarse-graining is also critical to tactical use of computational resources while simulating docking of a coating protein to the metal surface, as a step shows in our proposed workflow in Figure 5. This step was obviated in Ti-GelMA system because of ATRP bonding. Another possible reason for the observed result was the small training set size, as with a total of four peptides to allocate between the training and test set, a smaller than ideal training set resulted. Seen in PCA and re-emphasized in multiple regression, the model will predict a high bacterial load if most training set values are high, even if the ground truth result is not. PCA and multiple regression are likely incapacitated by differing trends in an extensive descriptor set. Alternative algorithms may be more capable of predictive discrimination. In comparison with PCA, autoencoders have been shown to depict clearer trends and subgroups when visualizing data while achieving comparable accuracy. Autoencoders can also be stacked into a deep neural net to increase predictive abilities at the cost of increased training times and computational demands [30,31]. Another strategy that could be useful is augmenting cheminformatic data prior to algorithmic processing by an autoencoder or an alternative learning method. [32,33] Regardless of whether or not autoencoders and data augmentation are used in the machine learning step, generation and processing of the structure-based molecular descriptors and correlation with bioactivity data could use our workflow (Figure 5).

## 4. Conclusions

In this study, we examined the potential of using structural information about GelMA and other proteins to predict their biocompatibility with implanted medical devices. We generated a workflow that fed structural data into a machine learning pipeline. It is possible that with further development many of these steps could be automated, generating software packages useful to the biomaterials and medical device community. From this investigation, we found that cheminformatic methods can be applied to methacrylated proteins to evaluate their suitability for coating titanium implants. Methacrylated proteins such as GelMA offer cell attachment sites for improved biocompatibility over uncoated titanium, while other methacrylated proteins that have biomaterial properties such as elasticity are important in the fabrication of stents for cardiovascular applications. Further development of this work will allow methacrylated protein-coated titanium implants, which will be less susceptible to biofilm formation and will show improvement in biocompatibility, leading to improvement in implant integration. Although the most immediate application of protein coatings of metal are medical devices, alternatives such as surface plasmon resonance, bio-organic solar cells or industrial biosynthesis are a few of many possible applications which could benefit from efficient testing and optimization of biocompatibility.

## Figures and Tables

**Table 1:** Calculated chemical descriptors.

Molecule	Mass (kDa)	pI seq	pI 3D	r g	Hydrodynamic Radius
Lysozyme (253L)	18.57	10.18	10.17	16.64	20.84
Fibrinogen (3GHG)	225.36	6.24	6.74	153.87	50.970001
Troponin-C (1NCX)	18.44	3.65	3.47	22.55	21.35
Collagen (1BKV)	7.96	12.6	10.29	24.8	13.92
Troponin-T (4Y99)	9.13	10.01	9.95	20.73	20.309999
Methacrylated-Troponin monomer	9.67	10.01	5.26	20.62	20.83
Methacrylated Troponin trimer	28.95	10.11	6.89	40.53	41.099998
GelMA trimer 210aa fragment	59.53	9.96	7.17	251	200.78999
GelMA trimer 30aa fragment	4.45	10.46	8.34	23.89	23.889999

**Table 2:** Additional structure-based molecular descriptors.

Molecule	Mobility	Net Charge	Dipole Moment	Zeta Potential
Lysozyme (253L)	17	10.58	310.07001	30.82
Fibrinogen (3GHG)	−50	−10.87	1442.53	−82.110001
Troponin-C (1NCX)	−66	−33.23	267.32999	−119.47
Collagen (1BKV)	15	3.9300001	1209.37	28.559999
Troponin-T (4Y99)	6.5	5.1100001	707.23999	11.75
Methacrylated Troponin monomer	−1.5	−0.07	631.57001	−2.73
Methacrylated Troponin trimer	−3.7	−5.2399998	722.01001	−6.3400002
GelMA trimer 210aa fragment	−0.12	−1.3200001	14932.37	−0.17
GelMA trimer 30aa fragment	5.5	3.01	597.71002	0

## Supplementary Materials

The following are available online at https://www.mdpi.com/2072-666X/11/1/87/s1.
