# Supplementary material for: Methacrylate Coatings for Titanium Surfaces to Optimize Biocompatibility
Source: Micromachines (Basel). 2020 Jan 13;11(1):87. doi: 10.3390/mi11010087 (PMC7019220; doi:10.3390/mi11010087)
Supplement: Supplementary file 1 [file micromachines-11-00087-s001.zip › Supplemental Files.pdf]

Supplemental Files

Descriptor Files

S1-S15

Descriptors calculated for proteins and methacrylated variants, the proteins are labelled with their PDB abbreviations.

Clinical and Lab Data

S16-S17

In vivo patient data from different protein coatings [20,21]

S18a&b

Data from antimicrobial peptides [19] cleaned for PCA using S19

Machine Learning Algorithm

S19

PCA script using Sci-kit learn with truncated and cleaned dataset
